# Exploring the Interrelationship Among Bone Status Indices: A Structural Equation Modeling Approach Reveals the Role of Osteocalcin as a Marker of Overall Bone Turnover

**DOI:** 10.3390/diagnostics16213634

**Published:** 2026-11-03

**Authors:** Martina Faraldi, Davide Ferrari, Massimo Locatelli, Eleonora Sabetta, Giuseppe Banfi, Giovanni Lombardi

**Affiliations:** 1 Laboratory of Experimental Biochemistry and Advanced Diagnostics, IRCCS Ospedale Galeazzi-Sant’Ambrogio, 20157 Milano, Italy; giovanni.lombardi@grupposandonato.it (G.L.); 2 SCVSA Department, University of Parma, 43121 Parma, Italy; davide.ferrari@unipr.it; 3 Laboratory Medicine Service, San Raffaele Hospital, 20132 Milano, Italy; locatelli.massimo@hsr.it (M.L.); sabetta.eleonora@hsr.it (E.S.); 4 Vita-Salute San Raffaele University, 20132 Milano, Italy; banfi.giuseppe@unisr.it; 5 Department of Athletics, Strength and Conditioning, Poznań University of Physical Education, 61-871 Poznań, Poland; lombardi@awf.poznan.pl

**Keywords:** bone status indices, bone metabolism, osteocalcin, osteoporosis, structural equation model, vitamin D

## Abstract

**Objective:** Bone status indices (BSIs) are key tools to monitor cell metabolic activity but lack specificity for clinical use. By applying a new statistical approach, this study aimed at highlighting the association between osteocalcin (OC), a regulatory molecule of bone turnover, often considered a putative biomarker of bone formation, and bone resorption markers like the β-isomerized C-terminal telopeptide of type I collagen (β-CTX-I) and urinary lysil-pyridinoline (uLysPYD), also investigating on the impact of vitamin D, age, and sex on their levels. **Methods:** Path analysis within the Structural Equation Modeling framework was applied to investigate relationships among BSIs. This model hypothesized OC as the outcome variable, affected by the direct effect of β-CTX-I, uLysPYD and 25-hydroxy-vitamin D [25-(OH)D] and by two 25-(OH)D indirect effects, through β-CTX-I and uLysPYD. **Results:** OC showed positive association with bone resorption markers, also when the model was run separately for sex and age groups. 25-(OH)D negatively associated with each bone biomarker. Age represented an effect modifier for 25-(OH)D relationship with both OC and β-CTX-I: the main differences were observed between the youngest (18–45) and the oldest cohorts (56–65 and >75) in both OC and β-CTX-I relationships with 25-(OH)D, and 46–55 and 66–75 groups in β-CTX-I and 25-(OH)D relationship. Both indirect effects were negative; the main effect of 25-(OH)D on OC was exerted through β-CTX-I. **Conclusions:** Rather than index of osteoblast activity, OC behaves as an overall turnover marker and possibly associated with resorption in elderly. The osteoclast-mediated bone resorption could impact OC serum levels, as it impacts β-CTX-I.

## 1. Introduction

Bone is a highly dynamic tissue subjected to a continuous remodeling throughout life [1]. Bone remodeling includes osteoclast-mediated resorption of the old matrix followed by osteoblast-mediated formation of new bone [2]. These metabolic processes are timely and spatially coupled within a functional unit called basic multicellular unit (BMU) [3]. Remodeling takes place constantly to preserve skeletal integrity, replace old bone (e.g., mechanically incompetent segments and old, less active osteocytes), maintain calcium and acid-base homeostasis, and regulate the release of growth factors buried in bone matrix [4]. Once linear growth and modeling are completed, the balance between formation and resorption changes according to life stage [1]. During physiological remodeling, after the achievement of peak bone mineral density (BMD) and during young adulthood, the rate of resorption equals the rate of formation, within each BMU, keeping the number of BMUs constant and bone mass stable. During the menopausal transition, and during aging, the equilibrium is shifted towards bone resorption while the number of BMUs increases [5].

The metabolic activity of bone cells can be evaluated, more or less directly, by measuring the bone status indices (BSIs) in biological fluids, providing a valuable and minimally invasive approach. BSIs are classified as markers of bone turnover, including procollagen type I N-propeptide (PINP), β-isomerized C-terminal telopeptide of type I collagen (β-CTX-I), hydroxyproline (HYP), pyridinoline (PYD), and deoxypyridinoline (DPD), bone cells’ enzymes, including total alkaline phosphatase (total ALP), bone-specific alkaline phosphatase (BALP), tartrate-resistant acid phosphatase 5b (TRACP5b), and regulatory molecules, as osteocalcin (OC), vitamin D and its metabolites [6]. However, within this category, it is important to distinguish between markers of bone formation, including PINP and BALP, and markers of bone resorption, including β-CTX-I, HYP, PYD, and DPD. OC is a bone-derived protein traditionally considered a marker of bone formation, but it may also reflect overall bone turnover because it can be released from the bone matrix during resorption. Furthermore, 25-(OH)D is considered a marker of vitamin D status and a regulator of bone metabolism, while ALP and TRACP5b may provide complementary information on bone cell activity [1]. Reflecting the metabolic activity of bone cells, BSIs allow the serial monitoring of bone processes under both physiological and pathological conditions, such as osteoporosis, as well as the assessment of compliance with and effectiveness of anti-osteoporotic therapies [7]. The primary goal in osteoporosis management is to prevent fractures, identified as fragility or low-trauma fractures, which represents the main cause of morbidity and mortality associated with osteoporosis [8]. Consequently, an early diagnosis is crucial. The diagnosis of osteoporosis is based on the measurements of BMD by dual-energy x-ray absorptiometry (DXA). However, early indicators of the disease are difficult to detect radiographically, and osteoporosis often remains clinically undiagnosed until a fracture occurs. Thus, combination of BMD measurement by DXA and BSIs detection would help in the early assessment of bone loss and fracture risk [9,10]. Rather than independent diagnostic tool, BSIs are valuable for predicting fracture risk and monitoring treatment response, as they reflect changes in bone turnover more rapidly than DXA [8]. The International Osteoporosis Foundation (IOF) and International Federation of Clinical Chemistry (IFCC) have recommended serum PINP and β-CTX-I as reference markers of bone formation and bone resorption, respectively, primarily for monitoring therapeutic response and adherence in osteoporosis management [11,12]. However, BSIs suffer of several issues that must be considered in the clinical contextualization of their measurement. The conventional distinction between bone formation and resorption markers is not entirely accurate, since each BSI can be produced or released during the opposite metabolic process. Thus, BSIs should be considered as biomarkers of the rate of bone turnover in general [13]. OC is a prime example of this complexity. As previously detailed, OC was traditionally considered a marker of osteoblastic activity, since it is synthetized by osteoblast and partly released into the circulation and partly incorporated into the mineralized matrix [14]. OC is already known to increase in the systemic circulation during bone resorption: the acidic environment created by bone-resorbing osteoclasts strips carboxyl groups from the protein, releasing undercarboxylated osteocalcin and fragments directly into circulation [15]. This evidence has suggested that circulating OC levels reflect both anabolic and catabolic processes and this dual origin has raised question on the role of OC as bone marker.

Moreover, BSIs are not entirely bone specific, as some of their components, such as collagen, are also present in other tissues. Nevertheless, given the greater contribution of bone mass relative to other tissues and the fact that BSIs mostly rely on components predominantly expressed in bone, the circulating levels of these analytes mainly reflect bone metabolic status [13,16].

Finally, the clinical implementation of BSIs requires more evidence on the multiple variables that can affect their circulating level, such as all pre-analytical and analytical variables [5].

Based on this contest, this study aimed at highlighting the network of relationship among BSIs, focusing on OC, one of the regulatory molecules of bone turnover, often considered a putative biomarker of bone formation, and its relationship with the bone resorption markers β-CTX-I and uLysPYD, considering the potential influence of endocrine variables (vitamin D) and constitutive variables (age and sex). To evaluate the hypothesized pattern of association and the relative influences existing between these different biomarkers, a path analysis within the Structural Equation Modeling (SEM) framework has been applied.

## 2. Materials and Methods

### 2.1. Dataset

**Figure 1:**
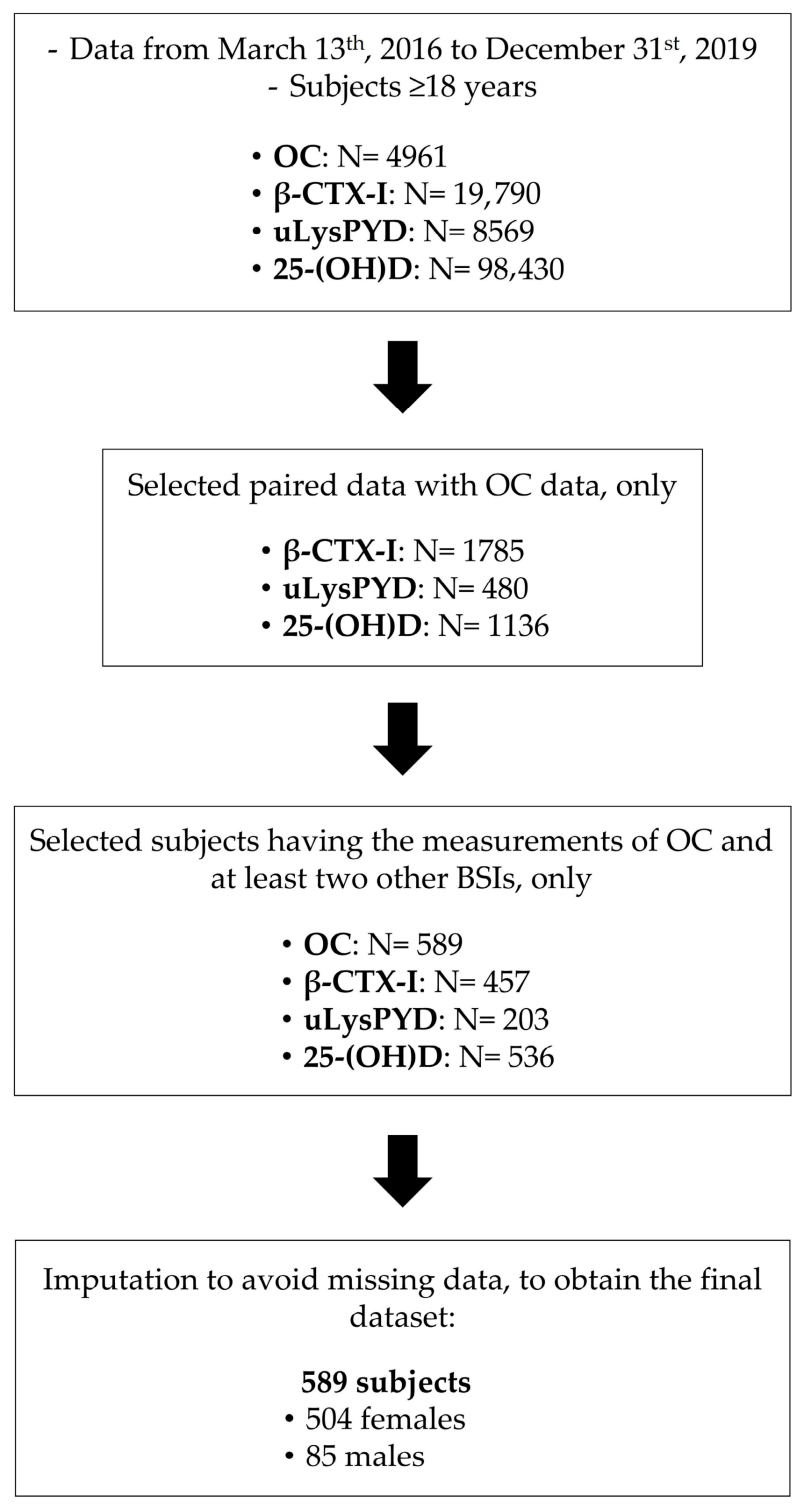
Flowchart of subject selection and study cohort definition.

This is a retrospective study on data collected by Laboratory Medicine Service of the San Raffaele Hospital (Milano, Italy), from 13 March 2016 to 31 December 2019, from male and female outpatients, aged 18 or older, who performed routine blood and/or urine sampling at IRCCS Ospedale San Raffaele (Figure 1).

Particularly, data on circulating levels of OC (μg/L), β-CTX-I (μg/L), uLysPYD (pmol/μmol uCr), and 25-hydroxy-colecalciferol (25-(OH)D) (μg/L) were considered. OC, β-CTX-I, and 25-(OH)D were measured in serum by electrochemiluminescence (ECLIA) on a Roche Cobas 8000 platform (Roche, Basel, Switzerland); uLysPYD was measured by High-Performance Liquid Chromatography (HPLC) in urine (Chromsystems, Gräfelfing, Germany) and concentration was normalized on the urinary level of creatinine (uCr). Urine samples were collected as first-morning urine specimens. The study included data from 13 March 2016 to 31 December 2019 from subjects older than 18 years (OC: N = 4961; β-CTX-I: nN = 19,790; uLysPYD: N = 8569; 25-(OH)D: N = 98,430). This dataset comprised a single measurement from each subject, with no repeated observations. For each analyte, only subjects having BSI data paired with OC data were considered. As a last step in defining the dataset, only subjects having the measurements of OC and at least two BSIs were included, to obtain a final dataset of 589 subjects. The dataset was imputed by impute() function, using nearest neighbor averaging method [17].

The study was conducted in accordance with the Declaration of Helsinki, and the protocol was approved by the Comitato Etico Ospedale San Raffaele, Milano, Italy (CET 242-2025) on 7 September 2025 and registered on Clinical Trial GOV (registration number: NCT07454252; registry name: OVAD) on 3 March 2026. This study was reported in accordance with the TREND checklist. Data were pseudonymized by assigning a unique identification code to each sample to encrypt any possibility of identification. Only authorized personnel were able to link the code to the corresponding individual. During the preparation of this manuscript, artificial intelligence OpenAI (GPT-6) was used exclusively to assist with grammatical correction. All text drafted with the aid of artificial intelligence was critically reviewed, corrected, and approved by the authors, who assume full responsibility for the accuracy and in-tegrity of the final manuscript.

### 2.2. Statistical Analysis

The sample size of the present study was calculated on G*Power v3.1.9.7 (Heinrich Heine University Dusseldorf, Dusseldorf, Germany) considering as primary endpoint the association of circulating levels of OC and vitamin D. Considering literature data on the relationship between OC and vitamin D [18] and assuming an effect size of 0.19, a type I error of 0.05, and a Power (1-error β) of 0.99, the resulting sample size was 493 subjects. To increase power analysis, 20% more subjects were included. All statistical analysis were performed on R 4.1.0 (R Foundation for Statistical Computing, Vienna, Austria) [19]. The sample size calculation was based on the association between OC and 25-(OH)D, rather than being specifically designed to determine the minimum sample size required for all parameters of the SEM or for the sex- and age-stratified analyses. Accordingly, subgroup analyses were considered exploratory, particularly in view of the limited number of male participant and the distribution of participants across age groups. Results were significant when *p* < 0.05. Descriptive statistical analysis was performed considering the entire sample and stratifying sample for sex and age. Five age groups (18–45, 46–55, 56–65, 66–75, >75) were defined to reflect major physiological stages of bone remodeling, ranging from young adulthood, menopausal transition, early post-menopause, older adulthood, to advanced age, which are characterized by progressive changes in bone turnover and skeletal homeostasis. Mean value, standard deviation, and quartiles were calculated. Normality of data was tested through a Quantile–Quantile plot, using qqnorm() function and, when necessary, data were transformed using natural log.

For each BSIs, a two-sided Z-test was used to compare the mean natural log-transformed values between males and females. The analysis of variance among age groups was performed using the anova() function, applied to the linear model with age as categorical independent variable. Post hoc pairwise comparisons were conducted using independent-samples *t*-tests, with Bonferroni correction applied to adjust for multiple comparisons. The analysis of variance among sex and age groups was performed using anova() function, applied to the linear model with categorical independent variables, followed by a post hoc analysis using the TukeyHSD() function, to account for multiple pairwise comparisons within the corresponding ANOVA model.

### 2.3. Structural Equation Model (SEM)

**Figure 2:**
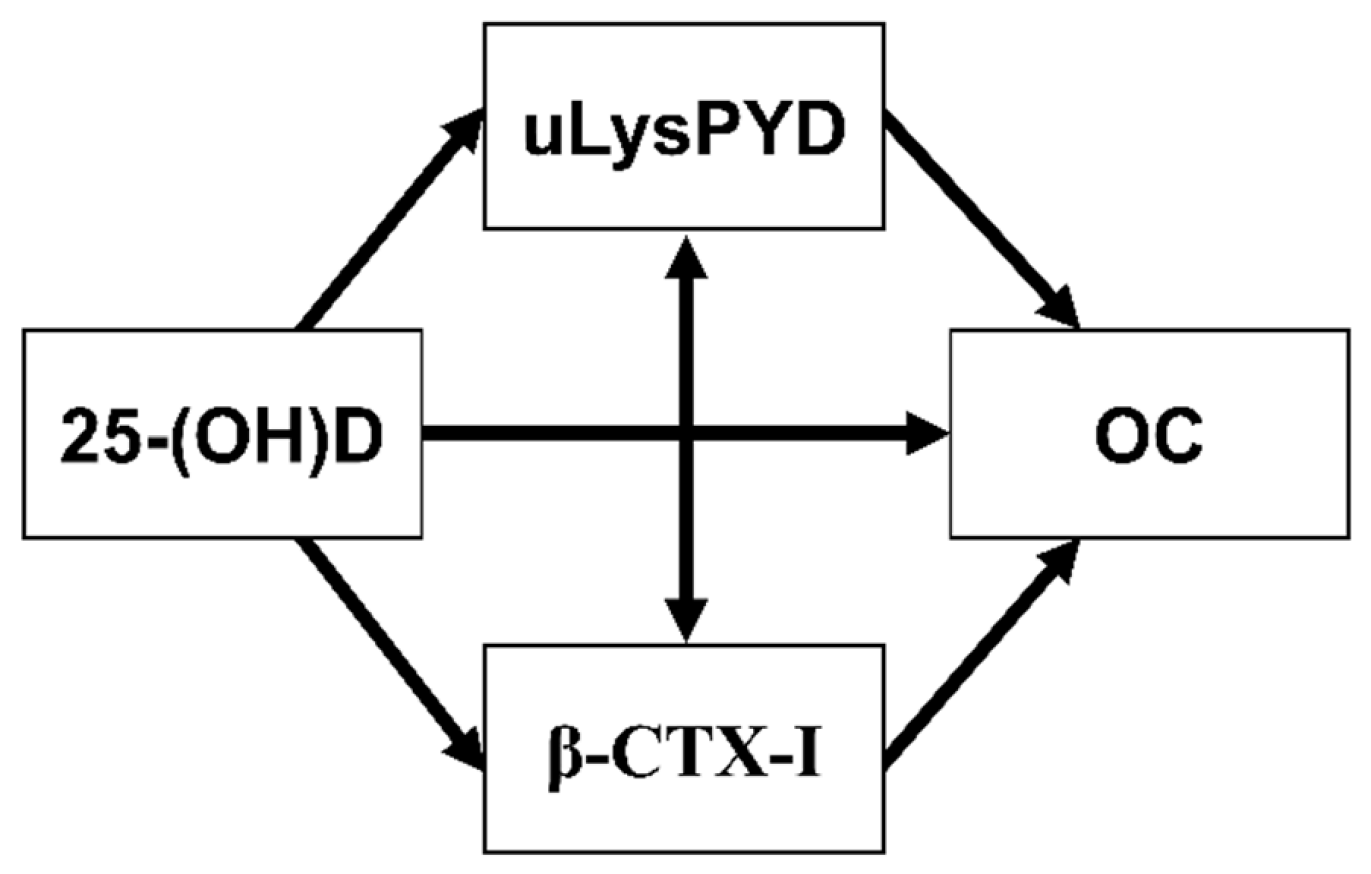
Path diagram describing the hypothesized relationships among osteocalcin (OC), β-isomerized C-terminal telopeptide of type I collagen (β-CTX-I), urinary lysyl-pyridinoline (uLysPYD), and 25-hydroxyvitamin D [25-(OH)D]. OC was specified as the outcome variable, whereas β-CTX-I, uLysPYD, and 25(OH)D were included as explanatory variables. The model estimates the direct effects of β-CTX-I, uLysPYD, and 25-(OH)D on OC, as well as the indirect effects of 25-(OH)D on OC mediated by β-CTX-I and uLysPYD. Covariance between β-CTX-I and uLysPYD was included to account for their shared variation as bone resorption markers. Single-headed arrows indicate regression (directed) paths, and double-headed arrows indicate covariances between variables.

To test the hypothesized pattern of associations among the different biomarkers, a path analysis was conducted within the SEM framework, and a basal model (MOD1) was developed (Figure 2).

This model comprises OC as the outcome variable and β-CTX-I, uLysPYD, and 25-(OH)D as explicative variables, and allowed the estimation of the direct effect of β-CTX-I, uLysPYD and 25-(OH)D, and two indirect effects of 25-(OH)D on OC, one through β-CTX-I and one through uLysPYD. Three models were also specified to test the effect of sex and age on BSI relationships: MOD2 adding sex and age as model covariates, MOD3 performing SEM analysis for males and females separately, and MOD4, performing SEM analysis by age groups. Age and sex were selected a priori as covariates because of their established relevance to bone metabolism and their availability in the retrospective dataset. Reduced model MOD1b and MOD2b were obtained by removing regression paths that did not reach statistical significance (*p*-Value > 0.05). Path selection was based exclusively on statistical significance and was not intended to demonstrate the absence of biologically plausible relationships when individual paths were non-significant. The resulting models were therefore interpreted as exploratory statistical models, with fit indices and estimated path coefficients reflecting statistical associations rather than necessarily representing biologically meaningful relationships.

SEM analysis was performed using the lavaan R package [20]. The hypothesized models were fitted by sem() function to estimate parameters. The structural equations parameters (regression coefficients (β) and residual variances) were computed by maximum likelihood estimates and the relative *p*-Values were evaluated by Z-tests (=estimate/standard error). *p*-Value < 0.05 in two-sided tests were considered statistically significant. In MOD3 and MOD4, comparisons of the estimates between males and females and among age groups were performed by regression coefficients differences using the output of the Z-tests.

Each SEM equation is associated to the R^2^, the amount of explained variance of a dependent variable by the model. Several fitting indices are provided by sem() function; model fitting procedure was evaluated with goodness-of-fit Chi-square statistic, Comparative Fit Index (CFI), and Standardized Root Mean Square Residual (SRMR). Non-significant χ^2^ test, CFI > 0.90, and SRMR < 0.05 were retained for “adequate approximation” fitting of the model to data. Specifically, the non-significant χ^2^ test suggests that the hypothesized model adequately reproduced the observed covariance structure; the CFI > 0.90 indicates excellent comparative fit, and the SRMR < 0.05 indicates minimal residual discrepancy between the observed and model-implied covariance matrices, supporting the adequacy of the final model.

## 3. Results

### 3.1. General Dataset Characteristics

This study includes data collected between the 13 March 2016 and the 31 December 2019. Restricting the analysis to subjects aged 18 years and older, 4961 data for OC, 19,790 data for β-CTX-I, 8569 data for uLysPYD, and 98,430 data for 25-(OH)D were obtained. For each BSI, only paired data with OC data were considered: 1785 for β-CTX-I, 480 for uLysPYD, and 1136 for 25-(OH)D. Only subjects having the measurements of at least two BSIs were included: 589 for OC, 457 for β-CTX-I, 203 for uLysPYD, and 536 for 25-(OH)D. The final dataset, imputed to avoid missing data (β-CTX-I: N = 132; uLysPYD: N = 386; 25-(OH)D: N = 53), included 589 subjects (504 females and 85 males) (Table 1).

No differences between females and males were observed for OC and β-CTX-I, while uLysPYD and 25-(OH)D levels were higher in females than in males (Table 1).

Considering the entire population, analysis by age groups showed significant variance for BSIs, except for uLysPYD (Table 2; Supplementary Figure S1).

More specifically, significant differences were observed between 46–55 and >75 groups for OC (*p* < 0.001) and β-CTX-I (*p* = 0.011), between 46–55 and 66–75 groups for β-CTX-I (*p* = 0.047), and between 18–45 and 56–65 groups for 25-(OH)D (*p* = 0.029) (Table 2; Supplementary Figure S1). Considering males and females stratified for age groups, the effect of age was significant on OC, β-CTX-I and 25-(OH)D levels, while the effect of sex was significant on uLysPYD and 25-(OH)D levels (Table 2). More specifically, in females, differences were observed in OC levels between the 46–55 and both 56–65 and >75 groups (*p* = 0.005 and *p* = 0.049, respectively), β-CTX-I levels between the 46–55 and >75 groups (*p* = 0.001), and uLysPYD levels between 18–45 and >75 groups (*p* = 0.030). Comparing the BSIs levels in males and females, differences were observed for uLysPYD in the >75 group (*p* = 0.015), and for 25-(OH)D in the 66–75 group (*p* = 0.019) (Table 2; Supplementary Figure S1).

### 3.2. MOD1: Basal SEM Analysis

MOD1 highlighted a positive association between OC and β-CTX-I (β = 0.45, *p* < 0.001), OC and uLysPYD (β = 0.26, *p* < 0.001), β-CTX-I and uLysPYD (β = 0.11, *p* < 0.010), and a negative association between OC and 25-(OH)D (β = −0.05, *p* = 0.031) and between β-CTX-I and 25-(OH)D (β = −0.28, *p* < 0.001), (Table 3).

The goodness of fit indices for the saturated MOD1 are shown in Supplementary Table S1. For exploratory purposes, MOD1b was obtained by removing the non-significant regression path from 25(OH)D to uLysPYD (Table 3), showing an adequate goodness of fit. For MOD1b the following improved fit indices were observed: χ^2^ = 2.527, *p* = 0.122; CFI = 0.998; SRMR = 0.026.

### 3.3. MOD2: SEM Analysis with Sex and Age as Covariates

MOD2 derived from MOD1 by adding sex and age as explicative variables that influence OC, β-CTX-I, uLysPYD, and 25-(OH)D. Estimated parameters revealed that sex was associated with uLysPYD (β = 0.11, *p* = 0.012) and 25-(OH)D (β = 0.24, *p* < 0.001) (Table 4).

Age was negatively associated with OC (β = −0.02, *p* = 0.018) and β-CTX-I (β = −0.01, *p* = 0.002), while positively associated with uLysPYD (β = 0.003, *p* = 0.015) (Table 4). OC was positively associated with β-CTX-I (β = 0.44, *p* < 0.001) and uLysPYD (β = 0.27, *p* < 0.001); β-CTX-I was positively associated with uLysPYD (β = 0.12, *p* < 0.001). Negative association was observed for 25-(OH)D with both OC (β = −0.05, *p* = 0.024) and β-CTX-I (β = −0.27, *p* < 0.001). In MOD2 also the relationship between uLysPYD and 25-(OH)D became significant (β = −0.06, *p* = 0.035).

By eliminating not statistically significant estimates, model MOD2b was generated (Table 4), showing the following improved fit indices: χ^2^ = 3.809, *p* = 0.432; CFI = 1; SRMR = 0.017 (Supplementary Table S1).

### 3.4. MOD3: Two-Group SEM ANALYSIS by Sex

To highlight differences between males and females, a two-group analysis by sex (MOD3) was performed (Table 5).

Both females and males showed positive associations between OC and β-CTX-I (β = 0.44, *p* < 0.001, and β = 0.49, *p* < 0.001, respectively) and between β-CTX-I and uLysPYD (β = 0.12, *p* < 0.001, and β = 0.07, *p* = 0.018, respectively), and a negative association between β-CTX-I and 25-(OH)D (β = −0.25, *p* < 0.001, and β = −0.44, *p* < 0.001, respectively). In females, a positive association was also observed between OC and uLysPYD (β = 0.30, *p* < 0.001) and a negative association between OC and 25-(OH)D (β = −0.05, *p* = 0.031). However, sex represented an effect modifier only for OC-uLysPYD relationship (*p* = 0.030) (Table 5, last column).

### 3.5. MOD4: Multi-Group SEM Analysis by Age

A multi-group analysis considering five age groups (18–45, 46–55, 56–65, 66–75, >75) was also fitted (MOD4) (Table 6).

Results showed positive associations between OC and β-CTX-I in all age groups, a positive association of OC and uLysPYD in all groups, except in the 18–45 group, and a negative association of OC and 25-(OH)D in the 56–65 and >75 groups. β-CTX-I was negatively associated (*p* < 0.05) with 25-(OH)D in the 56–65, 66–75, >75 groups and positively associated (*p* = 0.052) with 25-(OH)D in the 18–45 group. Association between uLysPYD and β-CTX-I was not significant only for the 18–45 group. uLysPYD−25-(OH)D relationship was the only one not being affected by age, since no significant estimate differences were observed among age groups (Supplementary Table S2).

### 3.6. Effect Decomposition of 25-(OH)D on OC

Basal SEM assumed 25-(OH)D acting on OC directly and indirectly through mediator variables (Table 7).

In MOD1b, both indirect effects of 25-(OH)D through β-CTX-I and uLysPYD were negative but significant only through β-CTX-I (β = −0.13, *p* = 0.001). After adjusting for sex and age (MOD2b), both indirect effects were negative and significant (β = −0.12, *p* < 0.001 and β = −0.02, *p* = 0.043, respectively).

Females and males showed negative indirect effects of 25-(OH)D on OC through β-CTX-I (β = −0.11, *p* < 0.001, and β = −0.22, *p* = 0.001, respectively), while non-significant indirect effects through uLysPYD were observed. Both indirect effects of 25-(OH)D on OC were not significant in the first two age groups, while in all the other groups (56–65, 66–75, >75), only the indirect effect of 25-(OH)D through β-CTX-I was significant.

## 4. Discussion

Bone formation and resorption are well reflected by BSIs that quantify osteoblasts and osteoclasts activity [1,5], as stated by IOF and IFCC for P1NP and β-CTX-I serum levels [11]. Although the utility of BSIs in monitoring osteoporosis treatments and in fracture risk prediction [21], the general increase of BSI levels in response to bone turnover reduces BSIs’ specificity and thus their routine clinical application [13,16].

To better describe the coupling among BSIs, this study investigated the relationships between bone remodeling-associated variables, such as serum levels of OC, β-CTX-I, and 25-(OH)D, and urinary levels of uLysPYD, using a path analysis within the SEM framework. SEM was selected to test the hypothesized pattern of relationships among observed biomarkers and to assess whether these relationships differed between population subgroups. In addition, it enabled the simultaneous estimation of all pathway coefficients, the quantification of both direct and indirect effects, and the evaluation of the overall consistency of the hypothesized model with the observed data using established model fit indices. The uncertainties regarding its role in bone turnover and implication in multiple physiological processes led us to consider OC as the outcome variable. OC is the main non-collagenous hydroxyapatite (HAP)-binding protein synthesized mainly by osteoblasts [22,23]. During bone formation, OC is produced by osteoblasts under the regulation of vitamin D. OC is mostly incorporated into the bone matrix prior to ossification and partly released into the circulation, making serum levels proportional to osteoblast activity [14]. Once released, circulating OC seems to explicate different endocrine functions depending on its carboxylation degree, mainly related to glucose metabolism, male fertility, bone–muscle crosstalk, modulation of parathyroid cell function and neuronal functioning [24,25,26,27]. However, bone resorption, as highlighted by several authors, can also affect OC serum levels, since OC incorporated in bone matrix could be released during this process [16]. As the result of both ongoing osteoblastic synthesis and osteoclastic breakdown of bone tissue, the relationship between OC and the other BSIs, and specifically with resorption markers, merits clarification.

β-CTX-I and uLysPYD were included as explanatory variables in our path model. Both are biomarkers of type I collagen catabolism resulting from osteoclast-mediated bone resorption. Specifically, β-CTX-I is generated through the enzymatic degradation of type I collagen by cathepsin K, whereas uLysPYD is a mature covalent collagen cross-link that stabilizes the three-dimensional fibrillar structure of collagen and is released during osteoclast-mediated collagen degradation before being excreted in the urine [16]. Although β-CTX-I and uLysPYD differ in their current clinical application, uLysPYD was included because it provides complementary information to β-CTX-I on collagen breakdown associated with bone resorption. As both markers are released from the bone matrix in proportion to the rate of bone resorption, β-CTX-I and uLysPYD were included in the model as explanatory variables, based on the hypothesis that they may exhibit distinct association with OC.

The model also includes 25-(OH)D, a key regulator of bone metabolism, playing a pivotal role in maintaining calcium and phosphate homeostasis and coordinating bone remodeling. Its active metabolite, 1,25-dihydroxyvitamin D (calcitriol), enhances the intestinal absorption of calcium and phosphate, thereby providing the mineral substrate required for bone mineralization. [28]. In addition, vitamin D directly influences osteoblast differentiation and function and indirectly regulates osteoclastogenesis by stimulating the expression of receptor activator of nuclear factor-κB ligand (RANKL) in osteoblasts and osteocytes [29]. Through these mechanisms, vitamin D contributes to the coupling of bone formation and bone resorption, ensuring the maintenance of skeletal homeostasis. Based on these biological mechanisms, vitamin D was included in the path model as an explanatory variable, as it may influence OC both directly and indirectly through its effects on osteoblast and osteoclast activity, thereby affecting the circulating and urinary biomarkers of bone remodeling: a direct effect of 25-(OH)D on OC and two indirect effects through β-CTX-I and uLysPYD were hypothesized.

Despite the advantages offered by BSI measurements, their interpretation requires consideration of biological sources of variability, particularly sex and age, which are among the main determinants of interindividual differences in BSI levels [1,8]. In this study, further path models were constructed accounting for age (18–45, 46–55, 56–65, 66–75, >75) and sex to highlight the changes in bone turnover rate and direction during life and in association with gender. Indeed, age- and sex-dependent changes in bone remodeling have been largely demonstrated [30]. In older individuals, the increased bone turnover is coupled with an imbalanced bone remodeling towards resorption, primarily due to the decrease of osteoblast differentiation and activity [1]. However, in females, a minimum bone turnover rate has been described during the premenopausal period (approximately between 35 and 45 years of age), while it reaches its peak in the early postmenopausal period and then slightly declines with aging, although it remains elevated. In males, instead, the rate of bone turnover peaks during adulthood (around 35–45 years of age), and then it slightly but constantly decreases [31].

When fitting the basic model (MOD1), a positive association was shown between OC and bone resorption markers, accordingly with the established coupling between bone resorption and formation [1]. This result was also confirmed when MOD1 was run adding age and sex as covariates (MOD2) and run separately for sex (MOD3) and age groups (MOD4). However, MOD3 showed that sex represented an effect modifier in the OC-uLysPYD relationship, as the association between these two biomarkers was not significant in males but significant in females. Age was considered in MOD4 as an effect modifier for OC-β-CTX-I association, suggesting that the strength of this relationship may differ across age groups. Nevertheless, this association remains positive for each age group. Although our findings reflect statistical associations rather than causal relationships, they suggest that, in older adults, circulating OC levels may be more closely related to bone resorption than to osteoblast activity. This observation is consistent with previous evidence challenging the conventional view of OC solely as a marker of bone formation [32,33].

25-(OH)D plays a regulatory role in bone remodeling; its serum levels were expected to associate with those of BSIs. In both MOD1 and MOD2, negative associations were observed between 25-(OH)D and each of the bone turnover markers. In MOD3, both OC and β-CTX-I were significantly associated with 25-(OH)D in females; however, sex did not represent an effect modifier. Contrarily, in MOD4, age acted as an effect modifier for both associations, and the main differences were observed between the youngest (18–45) and the oldest groups. Indeed, MOD4 highlighted a positive association of 25-(OH)D with both OC and β-CTX-I in the 18–45 group and a negative association in the groups 56–65, 66–75, and > 75, revealing an interesting age-dependent association [34]. Normally, this effect is evident in women, where the increase in bone turnover and the consequent increase of circulating level of bone biomarkers starts in perimenopause to continue into the postmenopausal period. In postmenopausal women, the increase in bone turnover results from both estrogens and 25-(OH)D deficiency, leading to an increase of osteoclast activity and bone resorption, and eventually to an increase of circulating levels of BSIs [30,35,36,37].

As previously described, MOD1 hypothesized the existence of multiple effects of 25-(OH)D on OC, composed of a direct and two indirect effects: through β-CTX-I and uLysPYD.

According to the four applied models, both indirect effects were negative, although only the indirect effect through β-CTX-I was significant. The association between 25-(OH)D and OC was predominantly represented by the indirect statistical path through β-CTX-I in the whole population, in males and females, and in the age groups 56–65, 66–75, and >75. These findings suggest that the relative contributions of both direct association and the indirect association involving β-CTX-I is a function of age. A possible interpretation for these statistical results may be that the observed indirect association of 25-(OH)D involving β-CTX-I may reflect the relationship between bone resorption and OC release from the bone matrix, as both processes may be associated with changes in bone turnover. In this context, our results support that age-related alterations in bone metabolism, potentially accompanied by lower 25-(OH)D levels, may be associated with concurrent changes in circulating OC and β-CTX-I. However, this interpretation remains speculative and requires confirmation in prospective studies.

Although SEM represents a useful approach for evaluating the hypothesized relationships among variables, the interpretation of the results depends on the validity of the assumed model. Our basic model is a saturated model, in which the number of observations equals the number of estimated parameters. Consequently, the fit indices indicate a perfect fit (i.e., zero discrepancy between the observed and model-implied covariance matrices). While this model provides information on the pattern of associations among the variables, its saturated nature limits its usefulness for predictive purposes. Therefore, alternative models (MOD1b and MOD2b) were generated to obtain more informative model fit statistics.

## 5. Conclusions

This study aimed to explore the relations among BSIs and endocrine factors that influence the release of bone biomarkers into the bloodstream using, for the first time, structural equation modeling. This approach allowed us to estimate the direct association between 25-(OH)D and OC and its indirect statistical association through the resorption markers, while exploring whether the observed relationships differed across age groups and between females and males.

Three main findings emerged: (1) OC was positively associated with bone resorption markers; (2) 25-(OH)D was similarly associated to OC and β-CTX-I; (3) the association between 25(OH)D and OC was predominantly represented by the indirect statistical path through β-CTX-I. These findings provide a more detailed description of the observed relationships among BSIs. However, given the design of the study and the assumptions underlying the specified path model, they should be interpreted as statistical associations rather than evidence of causal mechanisms or biological mediation.

Based on these findings, OC may be associated with bone resorption-related processes, showing a pattern of relationship with markers of osteoclast activity, including β-CTX-I and uLysPYD. This association appears to be more pronounced with aging and in the presence of reduced 25-(OH)D levels. This study supports the known uncertainty on the role of OC as a bone metabolism biomarker: while OC has traditionally been regarded as a marker of bone formation, reflecting osteoblast activity, it has been also considered an indicator of overall bone turnover, as it is released from matrix during bone resorption [16]. The absence of a reference marker of bone formation, such as PINP or BALP, is an important limitation. Although OC was positively associated with β-CTX-I and uLysPYD, the available data do not allow us to determine the relative contributions of bone formation and resorption to circulating OC levels. Incorporating a bone formation biomarker such as PINP or BALP as an explanatory variable in our model could provide additional insight into the role of OC as a bone biomarker.

Given the retrospective observational design, the study was based on a large sample size from routinely collected biochemical data, although heterogeneous due to the absence of restrictive inclusion and exclusion criteria. The unavailability of paired measurements of the analytes and the requirement for OC and at least two additional indices resulted in a missing value dataset and a final cohort of 589 subjects, including 504 females and 85 males. The study population was markedly imbalanced by sex, with substantially fewer males than females, consequently, estimates from the male subgroup and comparisons between sexes may be less precise and should be interpreted cautiously.

Considering the exploratory nature of the study, the limited clinical characterization of the study population prevented adjustment for several potentially relevant determinants of bone metabolism, including menopausal status, renal function, body mass index, season of sampling, vitamin D intake or supplementation, physical activity, and anti-osteoporosis medication use. Future prospective studies including comprehensive clinical characterization of the study cohort and a complete dataset, without missing data, are needed to improve the validity and confirm the BSIs’ observed relationships. Particularly, renal function represents an important factor to consider in the evaluation of bone metabolism, since impaired kidney clearance may affect circulating concentrations of both OC and β-CTX-I, potentially influencing their interpretation as markers of bone turnover [12].

Although SEM is well suited to test theory-driven biological hypotheses, the directionality of the specified relationships is based on prior biological knowledge rather than being inferred from the data alone, and alternative biologically plausible models may fit the data equally well. Therefore, the present findings should be interpreted as supporting the proposed network of direct and indirect associations rather than providing definitive evidence of causal relationships. Longitudinal and experimental studies will be necessary to confirm the causal nature of the observed associations.

## Figures and Tables

**Table 1:** Concentrations of OC, β-CTX-I, uLysPYD, and 25-(OH)D. Serum concentrations of OC, β-CTX-I, 25-(OH)D, and urinary concentration of uLysPYD determined in all samples, and samples grouped for sex. Data are reported as median (1st quartile–3rd quartile) and as mean ± standard deviation. Mean differences between females and males were analyzed through two-sided Z-test; *p* < 0.05 is considered significant. Significant *p*-Values are shown in bold. The analysis was performed on OC, β-CTX-I, uLysPYD, and 25-(OH)D concentrations in logarithmic scale.

	Total N = 589	Females N = 504	Males N = 85	*p*-Value
**OC (µg/L)**	22.2 (15.5–29.0) 23.80 ± 12.7	22.5 (15.5–29.6) 23.7 ± 11.4	20.1 (15.9–28.4) 24.6 ± 18.7	0.752
**β-CTX-I (µg/L)**	390 (238–537) 417 ± 252	392 (223–537) 413 ± 248	366 (284–528) 440 ± 279	0.410
**uLysPYD** **(pmol/umol CREAUR)**	11.26 (9.28–13.42) 11.66 ± 4.20	11.43 (9.55–13.60) 11.97 ± 5.56	10.38 (8.35–12.35) 10.88 ± 4.81	**0.019**
**25-(OH)D** **(µg/L)**	29.9 (22.2–39.7) 31.6 ± 15.2	30.5 (23.1–41.0) 32.6 ± 15.4	26.9 (16.8–34.3) 26.1 ± 12.8	**<0.001**

OC: osteocalcin; β-CTX-I: β-isomerized C-terminal telopeptide of type I collagen; uLysPYD: urinary lysil-pyridinoline; 25-(OH)D: 25-hydroxy-vitamin D.

**Table 2:** OC, β-CTX-I, uLysPYD, and 25-(OH)D concentrations in different age groups. Serum concentrations of OC, β-CTX-I, 25-(OH)D, and urinary concentration of uLysPYD in all samples, and samples grouped for sex, stratified for age. Data are reported as median (1st quartile–3rd quartile) and as mean ± standard deviation. Differences among age groups were analyzed through ANOVA F test with Tukey’s post hoc test; *p*-Value < 0.05 is considered significant. Significant *p*-Values are shown in bold. The analysis was performed on OC, β-CTX-I, uLysPYD, and 25-(OH)D concentrations in logarithmic scale.

	18–45 N = 42 M (13) F (29)	46–55 N = 118 M (18) F (100)	56–65 N = 149 M (16) F (133)	66–75 N = 165 M (16) F (149)	>75 N = 115 M (22) F (93)	Effect Size	*p*-Value
**OC (µg/L)**	21.2 (16.4–27.5) 22.2 ± 9.2	23.9 (17.9–31.0) 27.5 ± 17.1	22.3 (16.6–30.1) 23.9 ± 10.4	22.6 (15.2–29.7) 22.9 ± 10.1	19.5 (12.2–26.2) 21.5 ± 13.3	η^2^ age = 0.030	**0.001**
M	20.0 (14.8–31.27) 21.9 ± 8.4	21.0 (18.4–30.9) 33.1 ± 28.4	17.8 (11.8–21.3) 19.0 ± 12.3	20.9 (17.8–24.7) 23.1 ± 10.9	20.6 (13.1–27.9) 24.4 ± 20.3	η^2^ age = 0.030 η^2^ sex < 0.001 η^2^ interaction = 0.013	**0.001** 0.890 0.093
F	21.3 (16.7–25.9) 22.3 ± 9.6	24.4 (17.8–31.0) 26.5 ± 14.1	23.3 (17.1–30.4) 24.5 ± 10.1	23.2 (15.0–29.8) 22.9 ± 10.1	19.4 (12.2–26.2) 21.0 ± 11.7		
**β-CTX-I (µg/L)**	333 (270–460) 378 ± 211	444 (345–619) 489 ± 281	403 (263–531) 418 ± 217	360 (197–540) 401 ± 241	318 (159–492) 377 ± 278	η^2^ age = 0.033	**0.001**
M	321 (270–470) 381 ± 222	449 (336–615) 543 ± 381	308 (265–453) 370 ± 207	450 (283–553) 455 ± 254	362 (310–514) 429 ± 270	η^2^ age = 0.033 η^2^ sex = 0.002 η^2^ interaction = 0.005	**0.001** 0.292 0.558
F	337 (273–450) 378 ± 209	442 (346–619) 479 ± 260	412 (263–541) 423 ± 218	359 (194–538) 395 ± 240	302 (147–499) 367 ± 284		
**uLysPYD** **(pmol/µmol uCr)**	9.99 (8.50–11.10) 10.39 ± 4.32	11.15 (9.50–13.04) 11.54 ± 3.76	10.86 (9.32–12.79) 11.34 ± 3.67	11.53 (9.79–13.31) 11.68 ± 3.62	13.07 (9.60–16.21) 12.66 ± 5.53	η^2^ age = 0.011	0.162
M	9.98 (8.79–13.04) 11.17 ± 4.31	10.34 (8.97–12.50) 11.30 ± 3.91	10.07 (8.90–10.50) 10.07 ± 2.43	11.73 (10.53–12.18) 11.20 ± 2.82	8.46 (5.04–15.77) 10.74 ± 7.67	η^2^ age = 0.011 η^2^ sex = 0.003 η^2^ interaction = 0.019	0.155 **0.037** 0.060
F	9.99 (8.01–10.89) 10.53 ± 4.36	11.26 (9.67–13.06) 11.58 ± 3.75	11.12 (9.49–12.88) 11.49 ± 3.77	11.51 (9.71–13.30) 11.73 ± 3.70	13.13 (10.49–16.65) 13.11 ± 4.98		
**25-(OH)D** **(µg/L)**	26.0 (18.6–31.6) 26.8 ± 13.4	26.9 (21.3–33.6) 28.9 ± 13.7	33.1 (25.5–41.1) 33.5 ± 12.8	29.9 (21.8–40.9) 31.4 ± 14.3	31.8 (20.8–43.1) 33.9 ± 19.2	η^2^ age = 0.019	**0.022**
M	28.2 (16.8–31.3) 24.7 ± 96	23.3 (19.0–29.2) 24.2 ± 9.6	32.0 (26.0–41.3) 31.9 ± 12.4	18.3 (11.6–24.8) 21.9 ± 15.5	29.3 (12.1–36.9) 27.3 ± 14.3	η^2^ age = 0.019 η^2^ sex = 0.020 η^2^ interaction = 0.011	**0.020** <**0.001** 0.161
F	26.0 (18.8–36.7) 27.3 ± 14.9	27.2 (22.3–35.3) 29.7 ± 14.1	33.1 (25.5–41.1) 33.7 ± 12.8	30.5 (23.2–41.4) 32.4 ± 13.9	32.1 (22.8–46.1) 35.7 ± 20.9		

OC: osteocalcin; CTX-I: β-isomerized C-terminal telopeptide of type I collagen; uLysPYD: urinary lysil-pyridinoline; 25-(OH)D: 25-hydroxy vitamin D.

**Table 3:** MOD1 regression coefficients. SEM estimates, standard errors (SEs) and *p*-Values of MOD1 and MOD1b. MOD1b was obtained by excluding non-significant (*p* > 0.05) regression coefficients in MOD1 to enhance fitting of the model to data. Significant *p*-Values are reported in bold. Single tilde operator indicates regression coefficients, double tilde operator indicates covariances. The analysis was performed on OC, β-CTX-I, uLysPYD, and 25-(OH)D concentrations in logarithmic scale.

		MOD1			MOD1b	
Relationship	Estimate	SE	*p*-Value	Estimate	SE	*p*-Value
**OC~β-CTX-I**	0.45	0.02	**<0.001**	0.45	0.02	**<0.001**
**OC~uLysPYD**	0.26	0.04	**<0.001**	0.26	0.04	**<0.001**
**OC~25-(OH)D**	−0.05	0.02	**0.031**	−0.05	0.02	**0.032**
**β-CTX-I~25-(OH)D**	−0.28	0.05	**<0.001**	−0.25	0.05	**<0.001**
**uLysPYD~25-(OH)D**	−0.05	0.03	0.112			
**β-CTX-I~~uLysPYD**	0.11	0.01	**<0.001**	0.11	0.01	**<0.001**

OC: osteocalcin; β-CTX-I: β-isomerized C-terminal telopeptide of type I collagen; uLysPYD: urinary lysil-pyridinoline; 25-(OH)D: 25-hydroxy vitamin D.

**Table 4:** MOD2 and MOD2b regression coefficients. SEM estimates, standard errors (SEs) and *p*-Values of MOD2 and MOD2b, considering the variables sex and age as covariates. Significant *p*-Values are reported in bold. MOD2b was obtained by excluding non-significant (*p* > 0.05) regression coefficients in MOD2 to enhance fitting of the model to data. Single tilde operator indicates regression coefficients, double tilde operator indicates covariances. The analysis was performed on OC, β-CTX-I, uLysPYD, and 25-(OH)D concentrations in logarithmic scale.

		MOD2			MOD2b	
Relationship	Estimate	SE	*p*-Value	Estimate	SE	*p*-Value
**OC~β-CTX-I**	0.44	0.02	**<0.001**	0.44	0.02	**<0.001**
**OC~uLysPYD**	0.27	0.04	**<0.001**	0.27	0.04	**<0.001**
**OC~25-(OH)D**	−0.05	0.02	**0.024**	−0.05	0.02	**0.034**
**OC~Sex**	0.04	0.04	0.308			
**OC~age**	−0.02	0.001	**0.018**	−0.02	0.001	**0.019**
**β-CTX-I~25-(OH)D**	−0.27	0.05	**<0.001**	−0.27	0.05	**<0.001**
**β-CTX-I~Sex**	0.01	0.08	0.884			
**β-CTX-I~Age**	−0.01	0.002	**0.002**	−0.01	0.002	**0.002**
**uLysPYD~25-(OH)D**	−0.06	0.03	**0.035**	−0.06	0.03	**0.036**
**uLysPYD~Sex**	0.11	0.05	**0.012**	0.11	0.04	**0.007**
**uLysPYD~Age**	0.003	0.001	**0.015**	0.003	0.001	**0.014**
**25-(OH)D~Sex**	0.24	0.06	**<0.001**	0.24	0.06	**<0.001**
**25-(OH)D~Age**	0.002	0.002	0.262			
**β-CTX-I~~uLysPYD**	0.12	0.01	**<0.001**	0.12	0.01	**<0.001**
**Sex~~Age**	0.23	0.19	0.222			

SEM: structural equation model; OC: osteocalcin; β-CTX-I: β-isomerized C-terminal telopeptide of type I collagen; uLysPYD: urinary lysil-pyridinoline; 25-(OH)D: 25-hydroxy vitamin D.

**Table 5:** Regression coefficients for males and females (MOD3). SEM estimates, standard errors (SEs) and *p*-Values for males and females. The *p*-Value related to the difference between female and male estimates is also shown. Significant *p*-Values are reported in bold. Single tilde operator indicates regression coefficients, double tilde operator indicates covariances. The analysis was performed on OC, β-CTX-I, uLysPYD, and 25-(OH)D concentrations in logarithmic scale.

	MOD3						
Relationship	Females			Males			Difference *p*-Value
	Estimate	SE	*p*-Value	Estimate	SE	*p*-Value	
***OC~β-CTX-I***	0.44	0.02	**<0.001**	0.49	0.07	**<0.001**	0.484
***OC~uLysPYD***	0.30	0.04	**<0.001**	0.05	0.11	0.623	**0.030**
***OC~25-(OH)D***	−0.05	0.02	**0.031**	−0.08	0.08	0.332	0.590
***β-CTX-I~25-(OH)D***	−0.25	0.06	**<0.001**	−0.44	0.12	**<0.001**	0.158
***uLysPYD~25-(OH)D***	−0.04	0.03	0.154	−0.12	0.08	0.113	0.347
***β-CTX-I~~uLysPYD***	0.12	0.01	**<0.001**	0.07	0.03	**0.018**	0.180

SEM: structural equation model; OC: osteocalcin; β-CTX-I: β-isomerized C-terminal telopeptide of type I collagen; uLysPYD: urinary lysil-pyridinoline; 25-(OH)D: 25-hydroxy vitamin D.

**Table 6:** Regression coefficients for age groups (MOD4). SEM estimates, standard errors (SEs) and *p*-Values for age groups. Significant *p*-Values are reported in bold. Single tilde operator indicates regression coefficients, double tilde operator indicates covariances. The analysis was performed on OC, β-CTX-I, uLysPYD, and 25-(OH)D concentrations in logarithmic scale.

Relationship	18–45			46–55			56–65			66–75			>75		
	Estimate	SE	*p*-Value	Estimate	SE	*p*-Value	Estimate	SE	*p*-Value	Estimate	SE	*p*-Value	Estimate	SE	*p*-Value
**OC~β-CTX-I**	0.30	0.08	**<0.001**	0.50	0.05	**<0.001**	0.41	0.04	**<0.001**	0.46	0.04	**<0.001**	0.40	0.05	**<0.001**
**OC~uLysPYD**	0.03	0.11	0.782	0.42	0.09	**<0.001**	0.39	0.08	**<0.001**	0.37	0.07	**<0.001**	0.19	0.07	**0.011**
**OC~25-(OH)D**	0.13	0.09	0.139	0.03	0.05	0.571	−0.12	0.05	**0.022**	−0.02	0.04	0.608	−0.11	0.05	**0.038**
**β-CTX-I~25-(OH)D**	0.35	0.18	0.052	−0.17	0.12	0.161	−0.32	0.11	**0.004**	−0.42	0.09	**<0.001**	−0.37	0.11	**0.001**
**uLysPYD~25-(OH)D**	0.05	0.12	0.699	−0.08	0.06	0.185	−0.10	0.06	0.113	−0.06	0.04	0.206	−0.03	0.07	0.670
**β-CTX-I~~uLysPYD**	0.09	0.05	0.082	0.10	0.02	**<0.001**	0.10	0.02	**<0.001**	0.08	0.02	**<0.001**	0.19	0.04	**<0.001**

SEM: structural equation model; OC: osteocalcin; β-CTx-I: β-isomerized C-terminal telopeptide of type I collagen; uLysPYD: urinary lysil-pyridinoline; 25-(OH)D: 25-hydroxy vitamin D.

**Table 7:** Effect decomposition of 25-(OH)D on OC. SEM estimates, standard errors (SEs) and *p*-Values of direct and indirect, and total effect of 25-(OH)D on OC for each model: MOD1, MOD1b, MOD2, MOD2b, MOD3, and MOD4.

	**MOD1b**			**MOD2b**		
**Effect of 25-(OH)D**	**Estimate**	**SE**	***p*** **-Value**	**Estimate**	**SE**	***p*** **-Value**
**Direct**	−0.05	0.02	**0.032**	−0.05	0.02	**0.034**
**Indirect (through** **β** **-CTX-I)**	−0.13	0.02	**0.001**	−0.12	0.02	**<0.001**
**Indirect (through ULysPYD)**	−0.01	0.01	0.639	−0.02	0.01	**0.043**
**Total**	−0.19	0.04	**0.002**	−0.19	0.04	**<0.001**
	**MOD3 female**			**MOD3 male**		
**Effect of 25-(OH)D**	**estimate**	**SE**	***p*** **-Value**	**estimate**	**SE**	***p*** **-Value**
**Direct**	−0.05	0.02	**0.031**	−0.08	0.08	0.332
**Indirect (through** **β** **-CTX-I)**	−0.11	0.03	**<0.001**	−0.22	0.07	**0.001**
**Indirect (through uLysPYD)**	−0.01	0.01	0.161	−0.01	0.01	0.639
**Total**	−0.17	0.04	**<0.001**	−0.30	0.10	**0.002**
	**MOD4 18–45**			**MOD4 46–55**		
**Effect of 25-(OH)D**	**estimate**	**SE**	***p*** **-Value**	**estimate**	**SE**	***p*** **-Value**
**Direct**	0.13	0.09	0.139	0.03	0.05	0.571
**Indirect (through** **β** **-CTX-I)**	0.11	0.06	0.081	−0.08	0.06	0.164
**Indirect (through ULysPYD)**	0.001	0.01	0.822	−0.03	0.03	0.202
**Total**	0.24	0.10	**0.019**	−0.09	0.09	0.339
	**MOD4 56–65**			**MOD4 66–75**		
**Effect of 25-(OH)D**	**estimate**	**SE**	***p*** **-Value**	**estimate**	**SE**	***p*** **-Value**
**Direct**	−0.12	0.05	**0.022**	−0.02	0.04	0.608
**Indirect (through** **β** **-CTX-I)**	−0.13	0.05	**0.006**	−0.19	0.04	**<0.001**
**Indirect (through ULysPYD)**	−0.04	0.02	0.130	−0.02	0.02	0.220
**Total**	−0.29	0.08	**<0.001**	−0.21	0.09	**<0.001**
	**MOD4 > 75**					
**Effect of 25-(OH)D**	**estimate**	**SE**	***p*** **-Value**			
**Direct**	−0.11	0.05	**0.038**			
**Indirect (through** **β** **-CTX-I)**	−0.15	0.05	**0.002**			
**Indirect (through ULysPYD)**	−0.01	0.01	0.674			
**Total**	−0.27	0.07	**<0.001**			

SEM: structural equation model; OC: osteocalcin; CTx-I: β-isomerized C-terminal telopeptide of type I collagen; uLysPYD: urinary lysil-pyridinoline; 25-(OH)D: 25-hydroxy vitamin D.

## Data Availability

The original data presented in the study are openly available in Zenodo at https://doi.org/10.5281/zenodo.20021304 (31 August 2026).

## Supplementary Materials

The following supporting information can be downloaded at: https://www.mdpi.com/article/10.3390/diagnostics16213634/s1, Figure S1: Comparison of OC, β-CTX-I, uLysPYD, and 25-(OH)D levels. Comparison of OC, β-CTX-I, uLysPYD, and 25-(OH)D levels in the entire cohort, in males and females, in different age groups. All data are expressed as mean ± SD. Differences among groups were analyzed through ANOVA F test with Tukey’s post hoc test. The differences were considered significant when *p* < 0.05 (* *p* < 0.05, ** *p* < 0.01, *** *p* < 0.001, **** *p* < 0.0001); Table S1: Fitting indices and coefficients of determination (R2s) for hypothesized SEM; Table S2: Modification effect of age in MOD4. Differences among age group estimates and relative standard error. Significant difference estimates (*p* < 0.05) are represented in bold.

## Abbreviations

The following abbreviations are used in this manuscript:

25-(OH)D	25-hydroxy vitamin D
ALP	alkaline phosphatase
BALP	bone-specific alkaline phosphatase
BMD	bone mineral density
BMU	basic multicellular unit
BSIs	bone status indices
β-CTX-I	β-isomerized C-terminal telopeptide of type I collagen
DPD	deoxypyridinoline
DXA	dual-energy x-ray absorptiometry
FGF23	fibroblast growth factor 23
HAP	hydroxyapatite
HPLC	High-Performance Liquid Chromatography
HYP	hydroxyproline
IFCC	International Federation of Clinical Chemistry
IOF	International Osteoporosis Foundation
NTX-I	N-terminal telopeptide of type I collagen
OC	osteocalcin
PICP	procollagen type I C-propeptide
PINP	procollagen type I N-propeptide
PTH	parathyroid hormone
Q-Q plot	Quantile-Quantile plot
SEM	structural equation modeling
TRACP5b	tartrate-resistant acid phosphatase isoform 5b
uLysPYD	urinary lysil-pyridinoline
