# Supplementary material for: Exploring the Interrelationship Among Bone Status Indices: A Structural Equation Modeling Approach Reveals the Role of Osteocalcin as a Marker of Overall Bone Turnover
Source: Diagnostics (Basel). 2026 Nov 3;16(21):3634. doi: 10.3390/diagnostics16213634 (PMC13761942; doi:10.3390/diagnostics16213634)
Supplement: Supplementary file 1 [file diagnostics-16-03634-s001.zip › diagnostics-4569117-supplementary.pdf]

Table S1. Fitting indices and coefficients of determination (R2s) for hypothesized SEM.

	MOD1	MOD1b	MOD2	MOD2b	MOD3		MOD4				
					**Male**	**Female**	**18–45**	**46–55**	**56–65**	**66–75**	**>75**
***n* of observations**	589	589	589	589	85	504	42	118	149	165	115
***n* of free parameters**	10	9	21	17	28	28	70	70	70	70	70
**df**	0	1	0	4	0	0	0	0	0	0	0
**Chi square (*p*-value)**	0(0)	2.527(0.122)	0(0)	3.809(0.432)	0(0)	0(0)	0(0)	0(0)	0(0)	0(0)	0(0)
**CFI**	0	0.998	0	1	0	0	0	0	0	0	0
**SRMR**	0	0.026	0	0.017	0	0	0	0	0	0	0
**R2 OC**	0.600	0.596	0.600	0.603	0.638	0.454	0.375	0.677	0.639	0.653	0.558
**R2 β-CTX-I**	0.050	0.036	0.062	0.059	0.035	0.136	0.083	0.016	0.052	0.11	0.085
**R2 uLysPYD**	0.004		0.025	0.025	0.029	0.029	0.004	0.015	0.017	0.01	0.002
**R2 25-(OH)D**			0.026	0.024							

df: degrees of freedom; CFI: comparative fit index; SRMR: standardized root mean square residual; OC: osteocalcin; CTX-I: β-isomerized C-terminal telopeptide of type I collagen; uLysPYD: urinary Lysil-pyridinoline; 25-(OH)D: 25-hydroxy vitamin D.

Table S2. Modification effect of age in MOD4. Differences among age group estimates and relative standard errors. Significant difference estimates ($p < 0.05$) are represented in bold.

Relationship	18–45 vs. 46–55	18–45 vs. 56–65	18–45 vs. 66–75	18–45 vs. >75	46–55 vs. 56–65	46–55 vs. 66–75	46–55 vs. >75	56–65 vs. 66–75	56–65 vs. >75	66–75 vs. >75
					**Estimate Difference (SE)**					
**OC~β-CTX-I**	**−0.19 (0.08)**	−0.11 (0.09)	−0.16 (0.08)	−0.09 (0.09)	0.08 (0.06)	0.04 (0.06)	0.10 (0.07)	−0.05 (0.05)	0.02 (0.06)	0.06 (0.06)
**OC~uLysPYD**	**−0.38 (0.14)**	**−0.36 (0.14)**	**−0.33 (0.14)**	−0.16 (0.14)	0.02 (0.12)	0.05 (0.11)	**0.23 (0.12)**	0.03 (0.11)	0.20 (0.11)	0.18 (0.10)
**OC~25-(OH)D**	0.10 (0.10)	**0.25 (0.10)**	0.15 (0.10)	**0.25 (0.11)**	**0.15 (0.07)**	0.05 (0.07)	0.14 (0.08)	−0.10 (0.07)	−0.01 (0.08)	0.09 (0.07)
**β-CTX-I~25-(OH)D**	**0.52 (0.22)**	**0.68 (0.21)**	**0.78 (0.20)**	**0.73 (0.19)**	0.16 (0.16)	0.25 (0.15)	0.21 (0.16)	0.09 (0.15)	0.05 (0.16)	−0.04 (0.15)
**uLysPYD~25-(OH)D**	0.13 (0.14)	0.14 (0.14)	0.10 (0.13)	−0.08 (0.07)	0.01 (0.09)	−0.03 (0.08)	−0.05 (0.09)	−0.04 (0.07)	−0.07 (0.09)	−0.02 (0.08)
**β-CTX-I~~uLysPYD**	−0.01 (0.06)	−0.01 (0.06)	0.01 (0.26)	0.01 (0.26)	−0.01 (0.03)	0.03 (0.03)	−0.09 (0.05)	0.03 (0.27)	−0.08 (0.05)	**−0.11 (0.05)**

OC: osteocalcin; CTX-I: β-isomerized C-terminal telopeptide of type I collagen; uLysPYD: urinary Lysil-pyridinoline; 25-(OH)D: 25-hydroxy vitamin D

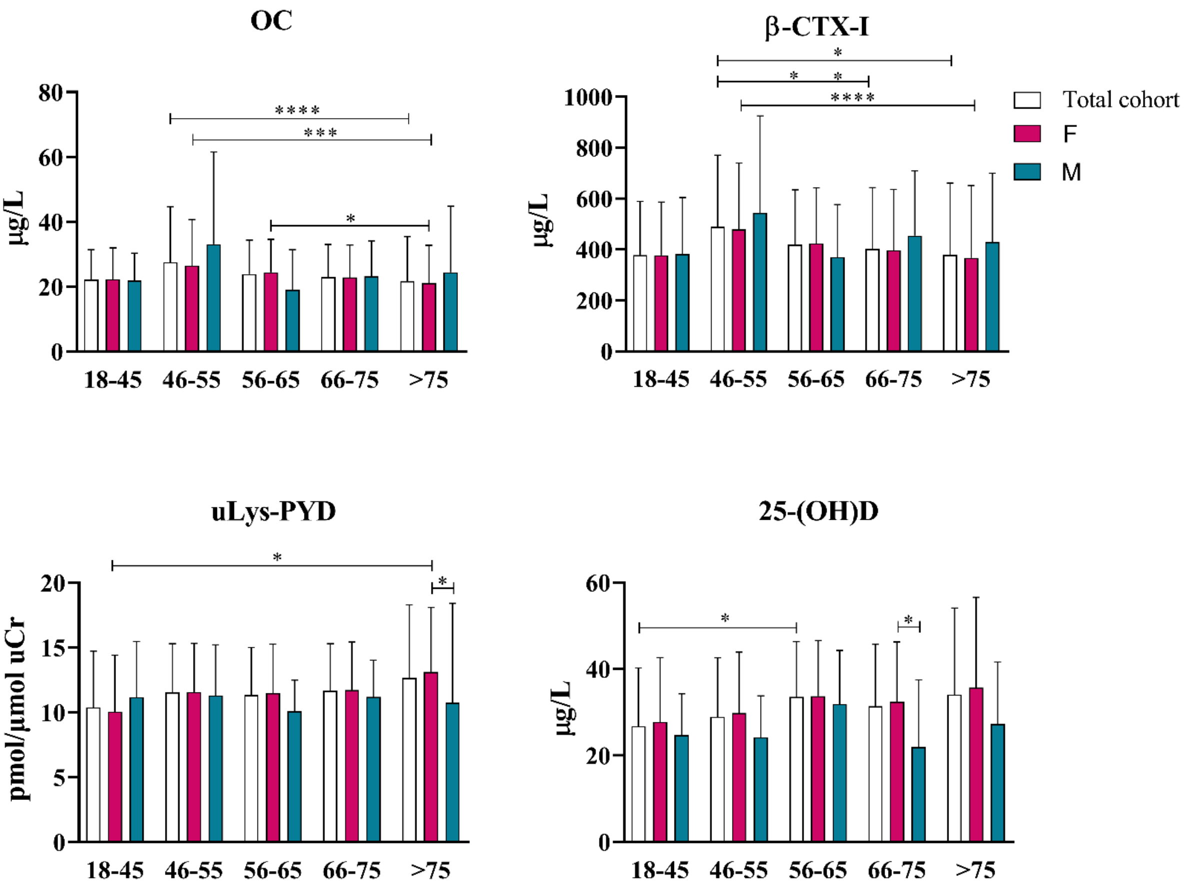

Figure S1. Comparison of OC, β-CTX-I, uLysPYD, and 25-(OH)D levels. Comparison of OC, β-CTX-I, uLysPYD, and 25-(OH)D levels in the entire cohort, in males and females, in different age groups. All data are expressed as mean ± SD. Differences among groups were analyzed through ANOVA F test with Tukey's post hoc test. The differences were considered significant when $p < 0.05$ (* $p < 0.05$, ** $p < 0.01$, *** $p < 0.001$ **** $p < 0.0001$).
